# Comparison of two doxycycline dosages on inflammatory and stress response in dogs naturally infected with *Dirofilaria immitis*

**DOI:** 10.1186/s13071-026-07505-y

**Published:** 2026-08-20

**Authors:** Daniel Julio Vera-Rodríguez, Rodrigo Morchón, Beatriz Regina Morales, José Alberto Montoya-Alonso, Elena Carretón, Noelia Costa-Rodríguez

**Affiliations:** 1 Internal Medicine, Faculty of Veterinary Medicine, Research Institute of Biomedical and Health Sciences (IUIBS), University of Las Palmas De Gran Canaria, Las Palmas De Gran Canaria, Spain; 2 Zoonotic Diseases and One Health Group, Laboratory of Parasitology, Faculty of Pharmacy, University of Salamanca, Salamanca, Spain

**Keywords:** *Dirofilaria immitis*, Canine heartworm disease, *Wolbachia pipientis*, Doxycycline, C-reactive protein, Inflammation, Anti-rWSP antibodies, Dogs

## Abstract

**Background:**

Canine heartworm disease, caused by *Dirofilaria immitis*, is characterized by a marked inflammatory response largely driven by its endosymbiotic bacterium *Wolbachia pipientis*. Doxycycline is routinely incorporated into treatment protocols to reduce *Wolbachia* burden and the associated pathology. Although the standard recommended dosage is 10 mg/kg every 12 h for 28–30 days, evidence comparing lower dosages under clinical conditions remains limited. This study aimed to compare the effects of two doxycycline dosages on inflammatory and immune markers in dogs naturally infected with *D. immitis* during the pre-adulticidal phase of treatment.

**Methods:**

Seventy-seven dogs naturally infected with *D. immitis* were randomly allocated to receive doxycycline at either 10 mg/kg (*n* = 39) or 5 mg/kg (*n* = 38) every 12 h for 30 days, in combination with a single oral dose of ivermectin (6 µg/kg) administered at treatment initiation. Serum concentrations of C-reactive protein (CRP) and cortisol were measured at baseline (day 0) and after treatment completion (day 30). Anti-recombinant *Wolbachia* surface protein (rWSP) antibody levels were determined by ELISA. Intra- and intergroup comparisons were performed using non-parametric statistical tests.

**Results:**

Both doxycycline regimens resulted in a significant reduction in CRP concentrations from day 0 to day 30 (*p* < 0.05), indicating a decrease in systemic inflammation. No significant differences were observed between dosages in the magnitude of CRP reduction. Cortisol concentrations showed a non-significant downward trend in both groups, while anti-rWSP antibody levels remained stable throughout the study period. No significant intergroup differences were detected for any evaluated parameter.

**Conclusions:**

Doxycycline administered at 5 mg/kg every 12 h for 30 days showed a comparable short-term biomarker response to the standard 10 mg/kg regimen during the pre-adulticidal phase. Lower doxycycline dosages may represent a potential alternative with possible benefits in terms of tolerance, compliance, and antimicrobial stewardship, although these aspects were not directly evaluated in the present study.

**Graphical Abstract:**

**Figure:**
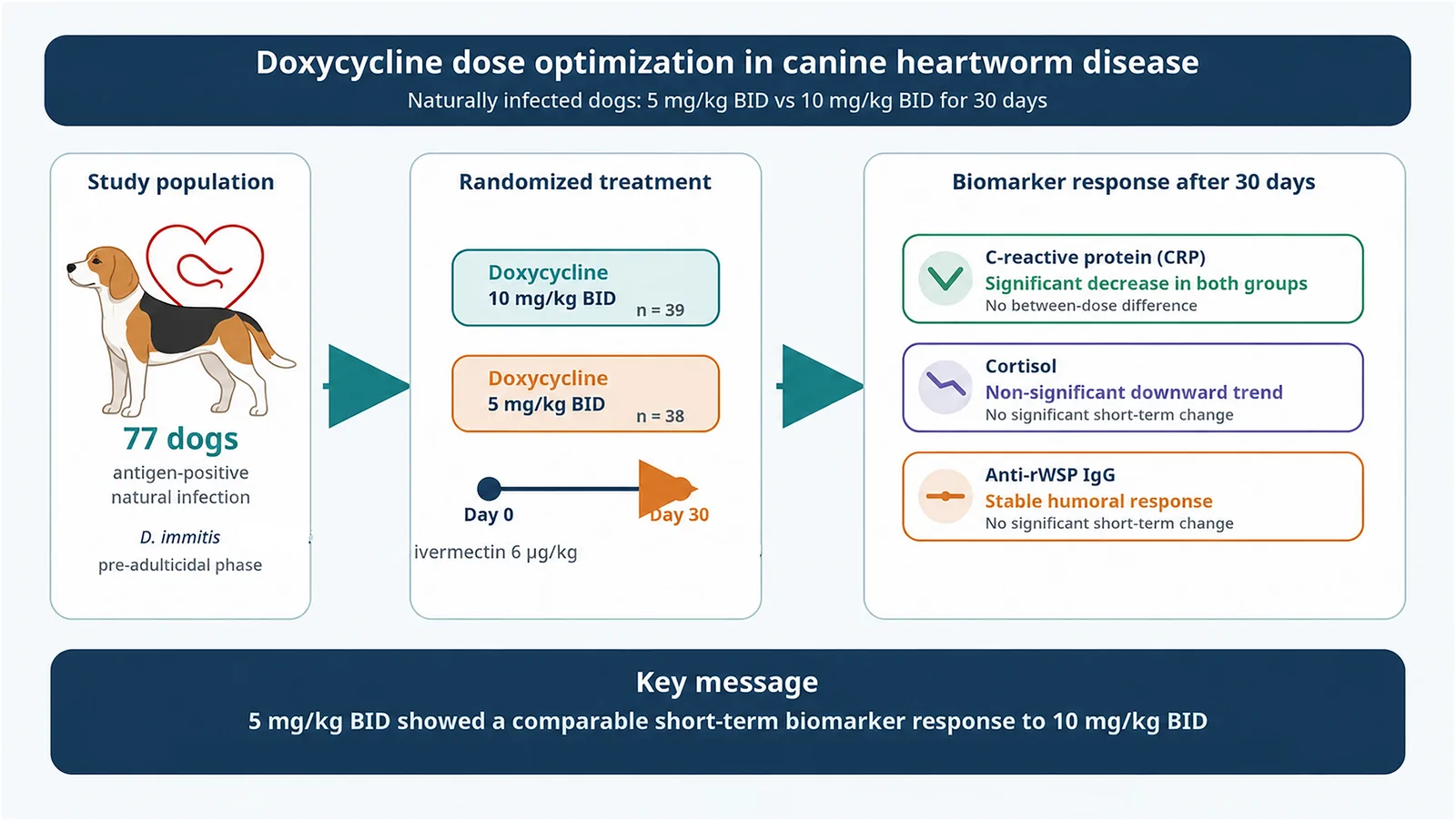

## Background

Canine cardiopulmonary dirofilariosis, commonly known as heartworm disease, is a parasitic infection caused by the filarial nematode *Dirofilaria immitis*. Dogs act as the main definitive hosts, although the parasite can also infect wild canids, cats, and humans, representing a relevant veterinary and zoonotic concern [1–3].

*Dirofilaria immitis* has a nearly worldwide distribution and has traditionally been associated with temperate and Mediterranean climates, where it is endemic in several European countries, as well as in large areas of the Americas. In recent years, however, its geographical range has expanded into regions with colder climates, likely driven by climate change, vector adaptation, and increased animal movement [4–8].

Most filarial nematodes, including *D. immitis*, maintain an obligate symbiotic relationship with the intracellular bacterium *Wolbachia pipientis* [9]. This endosymbiont is present in adult worms, larval stages, and microfilariae, and is vertically transmitted through the female reproductive tract. *Wolbachia* plays a central role in the pathogenesis of canine dirofilariosis, as its surface proteins and other bacterial components trigger a marked inflammatory and immune response in the host, thereby contributing to vascular, pulmonary, and systemic damage [10–12].

Consequently, infection with *D. immitis* may be associated with an acute-phase response, that develops during the course of infection, likely beginning with early parasite-induced vascular and inflammatory changes and becoming more evident as pulmonary injury progresses and patent infection is established. Among acute-phase proteins, C-reactive protein (CRP) has been consistently reported as a sensitive marker in dogs infected with heartworm [13–16]. In parallel, dogs with dirofilariosis may also exhibit increased cortisol concentrations, reflecting the combined effects of chronic inflammation, vascular injury, and the physiological stress associated with parasitism and clinical management [17–20].

In addition to these non-specific inflammatory markers, the host immune response against *Wolbachia* can be evaluated through the detection of antibodies directed against the recombinant *Wolbachia* surface protein (rWSP). Anti-rWSP antibodies are considered markers of exposure to, and immune activation against, the endosymbiotic bacterium and have been widely used to characterize the immunopathological response in *D. immitis* infection, although their kinetics during treatment may differ from those of classical acute-phase proteins [11, 12, 21, 22].

The incorporation of doxycycline into heartworm treatment protocols aims to reduce or eliminate *Wolbachia*, thereby attenuating the inflammatory response and minimizing treatment-associated pathology. Current international guidelines recommend doxycycline at a dosage of 10 mg/kg every 12 h for 30 days [23, 24]. Nevertheless, several studies have suggested that lower dosages, such as 5 mg/kg every 12 h, may achieve comparable reductions in *Wolbachia* burden while offering potential advantages in terms of tolerance, compliance, and antimicrobial stewardship [25–27].

Despite these findings, clinical data comparing the impact of different doxycycline dosages on inflammatory and immune markers during the pre-adulticidal phase of treatment in naturally infected dogs remain limited.

Therefore, the aim of the present study was to evaluate and compare the effects of two doxycycline dosages (10 mg/kg and 5 mg/kg, every 12 h for 30 days), in combination with orally administered ivermectin, on systemic inflammation and immune response in dogs naturally infected with *D. immitis*. Specifically, changes in CRP and cortisol concentrations were assessed as markers of the acute-phase and stress response, while anti-rWSP antibody levels were evaluated as indicators of the host immune response to *Wolbachia*, before treatment (day 0) and after completion of antibiotic therapy (day 30).

## Methods

The study included 77 dogs infected with *D. immitis* that attended the Veterinary Teaching Hospital of the University of Las Palmas de Gran Canaria between September 2020 and September 2023. For each animal a complete clinical record was obtained, including identification data, age, sex, breed, habitat, postal code, clinical signs (symptoms) at the start of treatment, echocardiographic findings, and routine hematological and biochemical analysis. The inclusion criteria were a positive *D. immitis* antigen test result and the absence of any prior specific treatment against the parasite.

Diagnosis was performed using a commercial immunochromatographic antigen detection test (Uranotest Dirofilaria^®^, Urano Vet SL, Barcelona, Spain). Dogs were randomly allocated into two treatment groups: Group A (*n* = 39), which received doxycycline at 10 mg/kg every 12 h, and Group B (*n* = 38), which received doxycycline at 5 mg/kg every 12 h.

In addition, all dogs received a single oral dose of macrocyclic lactones (ivermectin, 6 µg/kg) at treatment initiation.

Blood samples were collected from all dogs by cephalic venipuncture, and serum was obtained by centrifugation. Serum concentrations of CRP and basal cortisol were determined using a commercial immunochromatographic assay (VCHECK^®^, Bionote, Minnesota, USA). Reference values for healthy dogs, as provided by the manufacturer, were CRP < 10 mg/L and cortisol < 1.00 ug/dL. Measurements were performed at the time of diagnosis and initiation of treatment (day 0, D0) and after completion of the doxycycline regimen (day 30, D30).

Anti-rWSP antibody levels were determined using the same serum samples by means of a non-commercial enzyme-linked immunosorbent assay (ELISA), following previously described serological methods for anti-rWSP antibody detection, with minor modifications [21]. The rWSP used as coating antigen in the ELISA was produced according to the methodology described by Brattig et al. [28]. Briefly, 96-well microtiter plates were coated with 0.3 ug of rWSP per well and incubated overnight. After blocking, serum samples were added at a dilution of 1:20. Bound antibodies were detected using a horseradish peroxidase-conjugated anti-dog IgG (H + L) secondary antibody (Sigma-Aldrich, Spain), diluted 1:5000. The enzymatic reaction was developed using ortho-phenylenediamine (OPD) as the peroxidase substrate and allowed to proceed for 10 min before being stopped with 3 N sulfuric acid (${H}_{2}S{O}_{4}$). Optical density (OD) was measured at 492 nm using an Easy Reader microplate reader (Bio-Rad Laboratories, USA). The cutoff value for seropositivity (OD = 0.5) was established by calculating the mean OD obtained from 30 serum samples collected from clinically healthy canine blood donors (negative controls) living in a *D. immitis*-free area, plus three standard deviations (mean + 3 SD).

Data were analyzed using IBM SPSS Statistics 25.0 for Windows. Normality was assessed using the Shapiro–Wilk test. As data were not normally distributed, non-parametric tests were applied. Continuous variables were expressed as median and interquartile range (IQR). Treatment response was evaluated by calculating the absolute change (Δ = D30–D0) and the percentage reduction for each marker. Intragroup comparisons (day 0 vs. day 30) were performed using the Wilcoxon signed-rank test, while intergroup comparisons of Δ values were analyzed using the Mann–Whitney *U* test. Statistical significance was set at *p* < 0.05.

## Results

All dogs included in the study completed the 30-day doxycycline treatment and subsequently proceeded to adulticidal therapy. No clinically relevant adverse effects attributable to the treatment protocol, requiring hospitalization, pharmacological intervention, or additional clinical monitoring were observed during the 30-day doxycycline phase in either group.

At baseline (day 0), 48.0% of the dogs had CRP concentrations above the reference range (median: 10.00 mg/L; IQR: 10.00–18.20 mg/L), while elevated cortisol concentrations were detected in 55.84% of the animals (median: 1.13 ug/dL; IQR: 1.00–2.75 ug/dL).

When analyzed by treatment group, median CRP concentrations at day 0 were 12.10 mg/L (IQR: 10.00–28.00 mg/L) in Group A and 10.00 mg/L (IQR: 10.00–15.75 mg/L) in Group B. Baseline median cortisol concentrations were 1.00 ug/dL (IQR: 1.00–2.25 ug/dL) in Group A and 1.58 ug/dL (IQR: 1.00–2.83 ug/dL) in Group B. Baseline anti-rWSP antibody levels at day 0 were comparable between groups, with median OD values of 0.60 (IQR: 0.45–0.80) in Group A and 0.64 (IQR: 0.53–0.77) in Group B. No statistically significant differences were observed between groups at baseline for any of the evaluated parameters.

**Fig. 1:**
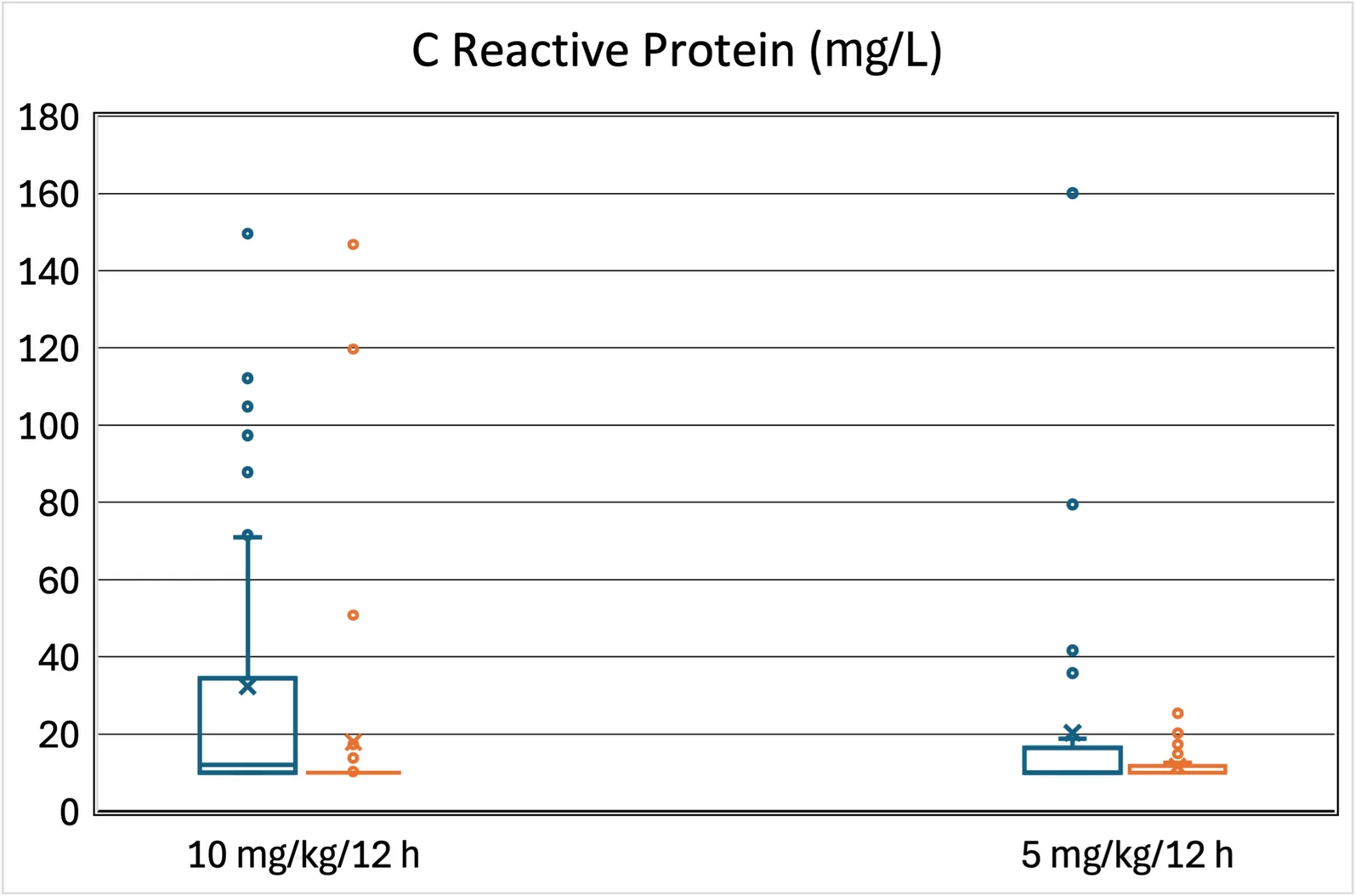
Pre- and post-treatment C-reactive protein (CRP) concentrations in dogs naturally infected with *Dirofilaria immitis*. Serum CRP concentrations (mg/L) measured at baseline (day 0, blue) and after completion of doxycycline treatment (day 30, orange) in dogs receiving doxycycline at 10 mg/kg (Group A) or 5 mg/kg (Group B) every 12 h for 30 days, in combination with ivermectin. In each box plot, the horizontal line represents the median, the box spans the interquartile range (25–75th percentiles), and the whiskers indicate the minimum and maximum values excluding outliers; individual outliers, when present, are shown as isolated points. The reference range for serum CRP was < 10 mg/L. A significant intragroup reduction in CRP concentrations was observed in both treatment groups (*p* < 0.05), with no significant differences between dosages

After completion of doxycycline treatment (day 30), CRP concentrations decreased in both treatment groups. Overall, 25.97% of the dogs still showed CRP values above the reference range. Median CRP concentrations at day 30 were 10.00 mg/L (IQR: 10.00–10.00 mg/L) in Group A and 10 mg/L (IQR: 10.00–11.28 mg/L) in Group B. Intragroup comparison revealed a statistically significant decrease in CRP concentrations from day 0 to day 30 in both groups (Wilcoxon signed-rank test, *p* = 0.00018) (Fig. 1).

**Fig. 2:**
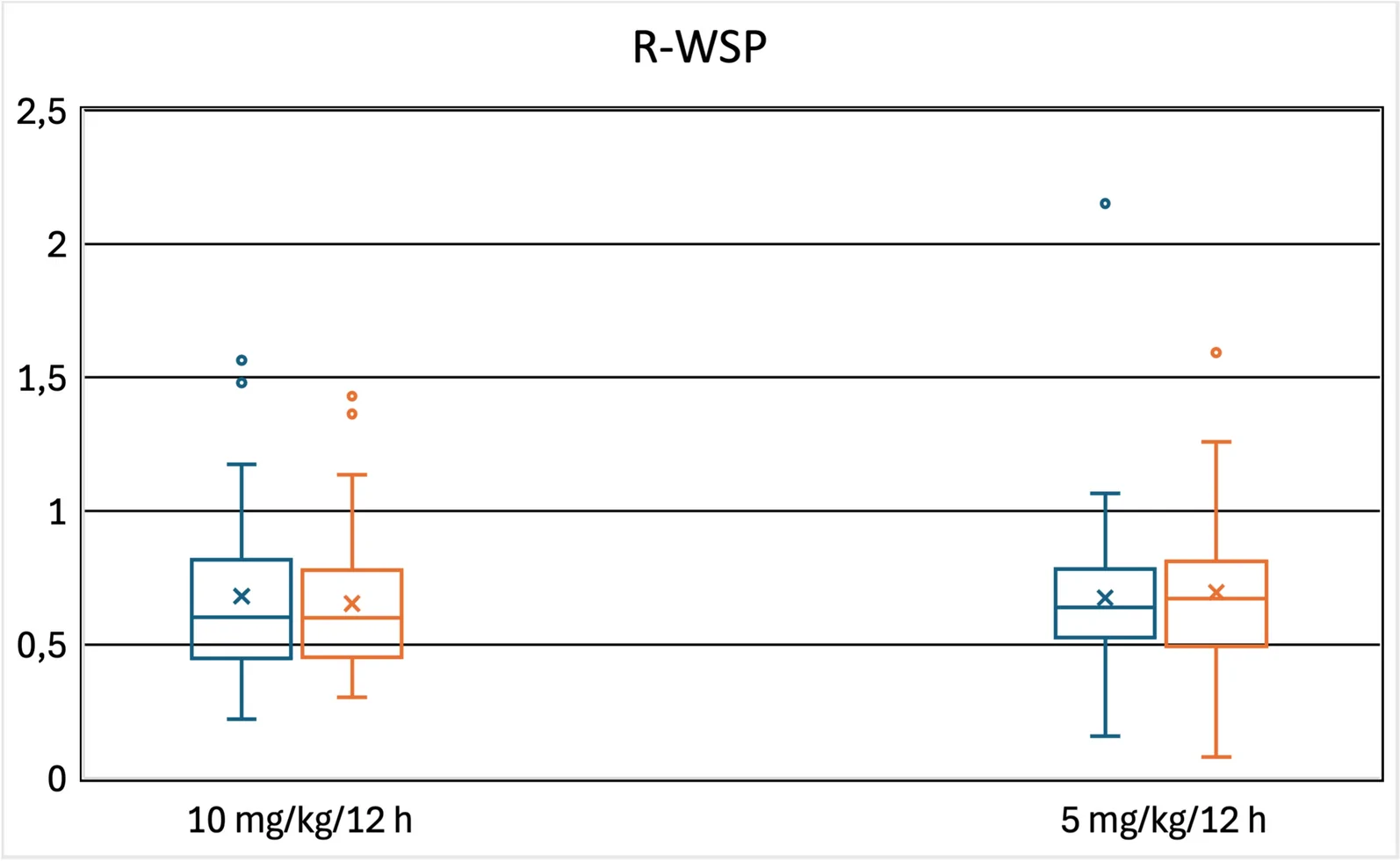
Pre- and post-treatment serum cortisol concentrations in dogs naturally infected with *Dirofilaria immitis*. Basal serum cortisol concentrations (µg/dL) measured at baseline (day 0, blue) and after completion of doxycycline treatment (day 30, orange) in dogs receiving 10 mg/kg (Group A) or 5 mg/kg (Group B) every 12 h for 30 days. In each box-and-whisker plot, the median is represented by the horizontal line, the box corresponds to the interquartile range (25–75th percentiles), and the whiskers denote the range of non-outlier values; outliers, when present, are shown as separate points. The reference range for serum cortisol was < 1 ug/dL. Although a decreasing trend was observed after treatment in both groups, no statistically significant intragroup or intergroup differences were identified

Cortisol concentrations showed a slight decrease after doxycycline treatment in both groups; however, intragroup comparisons did not reach statistical significance (*p* = 0.461). Median cortisol values at day 30 were 1.07 ug/dL (IQR: 1.00–2.31 ug/dL) in Group A and 1.00 ug/dL (IQR: 1.00–2.59 ug/dL) in Group B (Fig. 2).

**Fig. 3:**
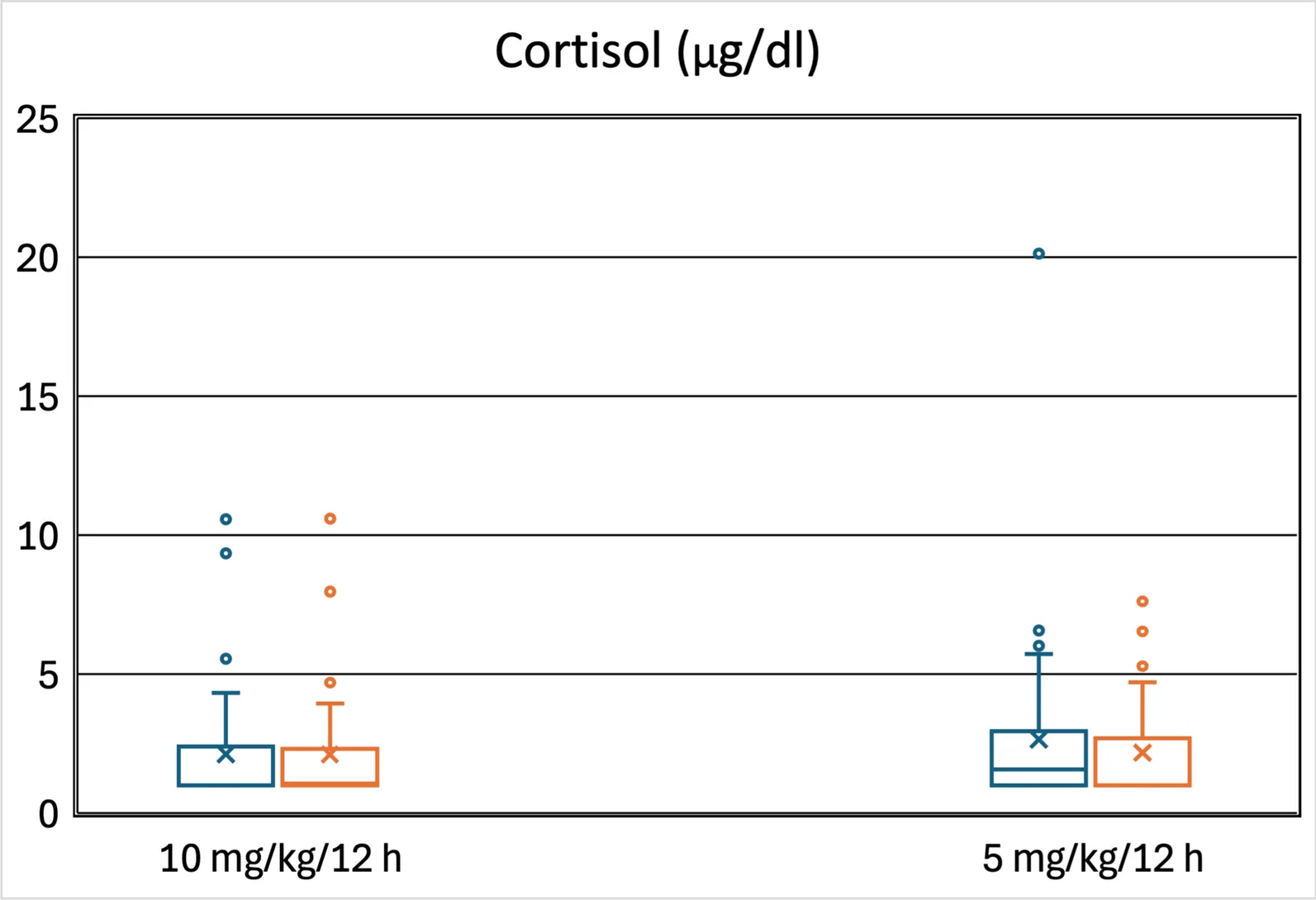
Anti-recombinant *Wolbachia* surface protein (rWSP) antibody levels before and after doxycycline treatment. Serum anti-rWSP IgG levels, expressed as optical density (OD) values, measured at baseline (day 0, blue) and after completion of doxycycline treatment (day 30, orange) in dogs treated with doxycycline at 10 mg/kg (Group A) or 5 mg/kg (Group B) every 12 h for 30 days. In each box plot, the central horizontal line indicates the median, the box represents the interquartile range (25–75th percentiles), and the whiskers extend to the minimum and maximum non-outlier values; outliers are displayed as individual data points. No significant intragroup or intergroup differences were observed, indicating stability of the humoral immune response against *Wolbachia pipientis* during the study period

Anti-rWSP antibody levels remained stable throughout the study period, with no significant intragroup differences detected between day 0 and day 30 in either treatment group (*p* = 0.124). Median values at day 30 were 0.60 (IQR: 0.46–0.77) in Group A and 0.67 (IQR: 0.50–0.80) in Group B. A slight numerical increase in median anti-rWSP antibody levels was observed in the 5 mg/kg group at day 30; however, this change was not statistically significant (Fig. 3).

The magnitude of change (Δ = D30−D0) in CRP, cortisol, and anti-rWSP antibody levels was calculated for each dog. Intergroup comparisons of Δ values showed no statistically significant differences between the two doxycycline dosages for any of the evaluated parameters (Mann–Whitney *U* test, *p* = 0.912 for CRP, *p* = 0.782 for cortisol, and *p* = 0.405 for rWSP antibodies).

## Discussion

The elimination of *W. pipientis* through the administration of doxycycline represents a cornerstone of the medical management of canine heartworm disease [9, 29, 30]. By reducing the burden of this bacterial endosymbiont, doxycycline contributes to attenuation of the inflammatory response and mitigation of treatment-associated pathology during adulticidal therapy. Although the efficacy of the recommended dosage of 10 mg/kg every 12 h has been well-documented [31–33], comparative data evaluating lower dosages under clinical conditions remain limited.

The elevated CRP concentrations observed at baseline in both groups are consistent with previous studies reporting increased acute-phase protein levels in dogs naturally infected with *D. immitis*, largely driven by the inflammatory response elicited by *W. pipientis* [13, 14, 16, 34]. In addition, reductions in CRP concentrations after 30 days of treatment with doxycycline combined with macrocyclic lactones have also been previously documented and attributed to decreased vascular inflammation secondary to *Wolbachia* elimination, as well as to the reduction of circulating microfilariae [15, 35].

In the present study, both doxycycline regimens (10 mg/kg and 5 mg/kg every 12 h for 30 days), administered in combination with ivermectin, resulted in a significant reduction in systemic inflammation, as assessed by CRP concentrations. Importantly, no significant differences were detected between dosages in the magnitude of CRP reduction, supporting the interpretation that the lower dosage produced a comparable short-term biomarker response to the standard regimen with respect to its anti-inflammatory effect during the pre-adulticidal phase of treatment. The comparable response observed with both doxycycline dosages suggests that a substantial decrease in *Wolbachia*-associated inflammatory stimuli may be achieved even at lower antibiotic concentrations, in line with experimental and clinical evidence supporting dose-optimization strategies [25, 27, 33].

Although the reduction in CRP observed after doxycycline treatment suggests attenuation of systemic inflammation during the pre-adulticidal phase, these findings should not be interpreted as direct evidence of reduced lung pathology associated with adult heartworm death. The biomarkers evaluated in this study are indirect indicators and do not specifically assess pulmonary injury during adulticidal treatment. Therefore, the possible relationship between these short-term changes and subsequent pulmonary pathology remains uncertain. Recent data from experimentally infected dogs showed that doxycycline administered at 5, 7.5, or 10 mg/kg BID, in combination with ivermectin, significantly reduced *Wolbachia* levels in adult worms at both day 30 and day 60, with no statistically significant differences among dosage groups [27]. However, those findings should be interpreted cautiously. At day 30, *Wolbachia* DNA remained detectable in whole blood in two of five dogs receiving 5 mg/kg BID, whereas all dogs receiving 7.5 or 10 mg/kg BID were negative. Moreover, *Wolbachia* levels in adult worms decreased further between day 30 and day 60 in all dosage groups, supporting the relevance of the 1-month waiting period before adulticidal therapy. Immunohistochemistry also showed persistent WSP staining in treated worms, although with an apparent reduction after treatment and further reduction by day 60. Therefore, while these data support the investigation of lower-dose doxycycline protocols, they do not establish biological equivalence between 5 mg/kg BID and 10 mg/kg BID regimens.

With respect to cortisol, the results of the present study revealed elevated concentrations in both groups, indicating the presence of a systemic stress response. These findings are consistent with previous studies evaluating the impact of parasitic infections in animals and humans, in which a positive association between cortisol concentrations and the presence of virulent parasites has been reported [17, 36, 37].

Although cortisol concentrations tended to decrease following doxycycline treatment in both groups, these changes did not reach statistical significance. This result is not unexpected, given the multifactorial regulation of cortisol secretion and its marked sensitivity to non-specific stressors, including handling, clinical procedures and environmental factors. Moreover, persistent vascular damage and/or the continued presence of adult parasites may contribute to sustained activation of the hypothalamic–pituitary–adrenal axis, even after partial attenuation of the inflammatory response [19, 38]. Consequently, cortisol should be interpreted as a complementary and non-specific marker rather than as a direct indicator of treatment efficacy in canine heartworm disease.

Regarding the immune response against *Wolbachia*, anti-rWSP antibody levels remained relatively stable throughout the study period and did not differ between treatment groups. The slight numerical increase in anti-rWSP values observed in the 5 mg/kg group at day 30 should be interpreted cautiously, as no significant intragroup or intergroup differences were detected. This finding may reflect the persistence and delayed kinetics of humoral responses, rather than a meaningful increase in *Wolbachia*-associated immune stimulation. This observation supports the concept that anti-rWSP antibodies primarily reflect prior exposure and immune sensitization rather than short-term variations in bacterial burden. Unlike acute-phase proteins, humoral immune markers often exhibit delayed or prolonged kinetics and may persist beyond the period of antibiotic administration [21, 26]. Similar findings have been reported in previous studies, in which anti-rWSP antibody levels remained detectable after doxycycline treatment at different dosages. This persistence has been attributed to the release of *Wolbachia* antigens following the death of microfilariae and larval stages induced by the initial administration of macrocyclic lactones [26]. Therefore, the absence of significant changes in anti-rWSP levels does not contradict the anti-inflammatory effect observed for CRP but rather underscores the distinct biological significance of these biomarkers.

From a clinical perspective, the absence of significant differences in the short-term inflammatory response between doxycycline dosages may be relevant when considering dose optimization in heartworm treatment protocols. Since 5 mg/kg every 12 h is within the dosage range commonly used for doxycycline in other veterinary indications, these findings raise the possibility that the higher regimen traditionally recommended for canine heartworm disease may not always be necessary to achieve short-term anti-inflammatory effects during the pre-adulticidal phase. However, extrapolation from other bacterial or rickettsial infections should be made with caution. In such infections, the target organisms are generally located in the host’s blood or tissues, where doxycycline exposure can be more directly inferred from systemic drug concentrations. In contrast, *W. pipientis* resides within *D. immitis* tissues, predominantly in the lateral cords of adult worms. Therefore, doxycycline must first be ingested by the parasite, absorbed, and distributed within worm tissues before reaching its bacterial target. Consequently, *Wolbachia* organisms may not be exposed to doxycycline concentrations equivalent to those achieved by bacteria located in the host circulation or tissues, and higher systemic plasma concentrations could theoretically be required to attain optimal intraparasitic exposure. Specific tissue distribution or pharmacokinetic studies in doxycycline-exposed heartworms would be needed to clarify this issue.

In practical terms, the use of a lower dosage may offer potential advantages, including improved gastrointestinal tolerance, enhanced owner compliance, reduced treatment costs, and a more judicious use of antimicrobials while showing a similar short-term biomarker profile in the variables evaluated here [39, 40]. These considerations are particularly relevant for doxycycline, a drug with well-recognized gastrointestinal adverse effects in dogs, especially when administered at relatively high dosages and for prolonged periods. In canine heartworm protocols, the traditionally recommended regimen of 10 mg/kg every 12 h for 28–30 days represents a higher and more sustained exposure than that commonly used for many other veterinary indications, whereas the 5 mg/kg every 12 h dosage evaluated here is closer to standard therapeutic use. Nevertheless, these potential advantages remain theoretical in the context of the present study, as the present study did not assess gastrointestinal adverse effects, treatment adherence, intraparasitic doxycycline concentrations, or long-term clinical outcomes. This may be especially relevant in endemic areas, where heartworm disease is frequently diagnosed and treatment strategies must balance efficacy, safety, and sustainability.

Although no significant between-group differences were detected in the biomarkers evaluated here, this should not be interpreted as proof of biological or clinical equivalence between doxycycline dosages. Numerical differences in *Wolbachia* reduction within adult worms may still be clinically relevant, but this question cannot be resolved from the present data, as pulmonary histopathology and other direct measures of treatment-associated lung injury were beyond the scope of this study.

The interpretation of the biomarker response observed in the present study should also consider that doxycycline may exert effects beyond *Wolbachia* depletion. In addition to its antimicrobial activity, doxycycline has recognized anti-inflammatory and immunomodulatory properties, including modulation of inflammatory mediator production and matrix metalloproteinase activity [41, 42]. Therefore, the reduction in CRP observed in both treatment groups cannot be attributed exclusively to decreased *Wolbachia* burden, but may reflect the combined effects of anti-*Wolbachia* activity, direct anti-inflammatory actions of doxycycline, ivermectin administration, and the evolving host–parasite interaction during the pre-adulticidal period.

Several limitations of the present study should be acknowledged. First, the lack of direct quantification of *Wolbachia* burden, such as molecular detection of bacterial DNA, limits the ability to establish a direct correlation between biomarker changes and bacterial clearance. Second, the follow-up period was intentionally restricted to the 30-day doxycycline phase in order to assess short-term changes before adulticidal therapy; however, this design precluded evaluation of later timepoints, such as day 60 or day 90, which may better reflect longer-term inflammatory and immunological dynamics and potential changes associated with adult worm death. Finally, although the sample size was sufficient to detect within-group changes, larger cohorts may be required to detect subtle differences between dosages. This reinforces the need for future studies, including longer follow-up timepoints, particularly days 60 and 90, direct quantification of *Wolbachia* burden, assessment of intraparasitic doxycycline exposure, and evaluation of clinical outcomes during and after adulticidal therapy.

In conclusion, the results of this study indicate that doxycycline administered at 5 mg/kg every 12 h for 30 days showed a comparable short-term biomarker response to the standard 10 mg/kg regimen in dogs naturally infected with *D. immitis* during the pre-adulticidal phase. These findings support further evaluation of lower doxycycline dosages as a potential alternative within heartworm treatment protocols and highlight the need for additional studies aimed at refining dose-optimization strategies in canine dirofilariosis.

## Conclusions

Doxycycline administration at both 10 mg/kg and 5 mg/kg every 12 h for 30 days resulted in a significant reduction in systemic inflammation, as evidenced by decreased C-reactive protein concentrations, in dogs naturally infected with *D. immitis*. No significant differences were observed between dosages in the magnitude of this effect. In contrast, cortisol concentrations and anti-rWSP antibody levels did not show significant short-term changes following antibiotic treatment.

These findings indicate that a lower doxycycline dosage (5 mg/kg every 12 h) showed a comparable short-term biomarker response to the standard regimen during the pre-adulticidal phase of heartworm treatment. The use of reduced dosages may be considered a potential alternative in this setting, with possible benefits in terms of tolerance, compliance, and antimicrobial stewardship, although these aspects were not directly evaluated in the present study.

## Data Availability

Data supporting the main conclusions of this study are included in the manuscript.
